# Clinical and Radiographic Characteristics as Predictive Factors of Swelling and Trismus after Mandibular Third Molar Surgery: A Longitudinal Approach

**DOI:** 10.1155/2018/7938492

**Published:** 2018-04-23

**Authors:** José Manuel Pérez-González, Vicente Esparza-Villalpando, Ricardo Martínez-Rider, Miguel Ángel Noyola-Frías, Amaury Pozos-Guillén

**Affiliations:** ^1^Department of Oral and Maxillofacial Surgery, Faculty of Dentistry, San Luis Potosi University, San Luis Potosí, SLP, Mexico; ^2^Engineering and Materials Science Postgraduate Program, San Luis Potosi University, San Luis Potosí, SLP, Mexico; ^3^Basic Sciences Laboratory, Faculty of Dentistry, San Luis Potosi University, San Luis Potosí, SLP, Mexico

## Abstract

**Introduction:**

Factors that contribute to swelling and trismus are complex, and they are originated by surgical trauma. The aim of the present study was to determine whether clinical and radiographic factors could predict the level of swelling and trismus after lower third molar surgery, through longitudinal approach.

**Methodology:**

A prospective longitudinal trial was carried out. Forty-five patients of both genders with clinical and radiographic diagnosis of asymptomatic mandibular impacted third molar and with no intake of analgesic or anti-inflammatory drugs 12 h prior to surgery were recruited and evaluated in a 72 h follow-up period. A mixed repeated measures model and backward and restricted maximal likelihood methods were used to analyze the data.

**Results:**

Male gender, body mass index (BMI), the relation to the lingual and buccal walls, and age were determinants for predicting postoperative swelling and for exerting a significant influence (*P* < 0.05).

**Conclusions:**

This study suggests the association of male gender, the relation to lingual and buccal walls, BMI, and age with measurement of swelling.

## 1. Introduction

Surgical extraction of mandibular third molars under local anesthesia involves the traumatic manipulation of bone, connective, and muscle tissues. Swelling, pain, and trismus are the principal postoperative signs and symptoms, which are caused mainly by tissue damage [1]. The effect of mandibular third molar surgery on the postoperative period in the majority of patients is marked by pain, swelling, and trismus, either alone or in combination [2]. Control of these conditions comprises an important factor for clinicians, because lower third molar surgery is one of the most common procedures carried out by oral and maxillofacial surgeons [3–7].

Factors that contribute to swelling and trismus are complex, and they are originated by surgical trauma [8, 9]. The control of swelling, pain, and trismus would be based on the understanding of the associated preoperative factors involved on the postoperative results. Previous reports of preoperative conditions related with swelling, pain, and trismus have included clinical and radiological factors [10], the difficulty of the procedure [11, 12], intraoperative factors [13, 14], and patients' characteristics [15]. These studies used a correlation between the different factors and the outcomes; the difficulty of this approach is that the clinical outcomes are dynamic and they change as a function of time. In transversal or punctual measurements, it is not possible to observe changes over time.

Considering a longitudinal approach, the aim of the present pilot study was to determine whether clinical and radiographic factors could predict the level of swelling and trismus after lower third molar surgery.

## 2. Materials and Methods

**Figure 1:**
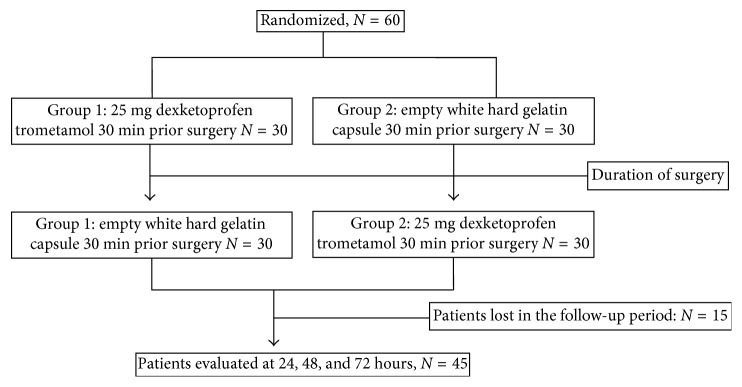
Flow chart of patient's distribution.

As part of previous study [4], the present prospective, longitudinal trial recruited 45 patients between 18 and 30 years old from the Department of Oral and Maxillofacial Surgery of the Faculty of Dentistry who had completed the 72-hour follow-up period. Briefly, the patients were assigned to two experimental groups. The first group received 25 mg of dexketoprofen trometamol 30 min before surgery and 1 placebo capsule immediately after the surgery. The second group received the placebo capsule 30 min before surgery and 25 mg of dexketoprofen trometamol immediately after the surgery. The local anesthesia was achieved by inferior alveolar nerve and lingual nerve blockade using lidocaine 2% with epinephrine 1 : 100,000 mL (FD ZEYCO, México), to remove one asymptomatic mandibular third molar. All the surgeries required mucoperiostical flap and osteotomy and were performed by the same surgeon. The patient distribution diagram is shown in Figure 1. The study was conducted in accordance with the Declaration of Helsinki, and the Institutional Ethics Committee approved the study design (CEI-FE-028-014). All participants were informed of the risks of oral surgery, and they signed a consent form.

Inclusion criteria were as follows: patients from both genders, with clinical and radiographic diagnosis of asymptomatic mandibular impacted third molar, and with no intake of analgesic or anti-inflammatory drugs 12 h prior to surgery. Exclusion criteria were the following: pregnant or breastfeeding patients, with the presence of systemic diseases such as diabetes, uncontrolled hypertension, or gastric ulcer, and patients with suspicion or evidence of narcotic or illicit drug use. Elimination criteria were all patients lost in the follow-up period (72 hours).

**Figure 2:**
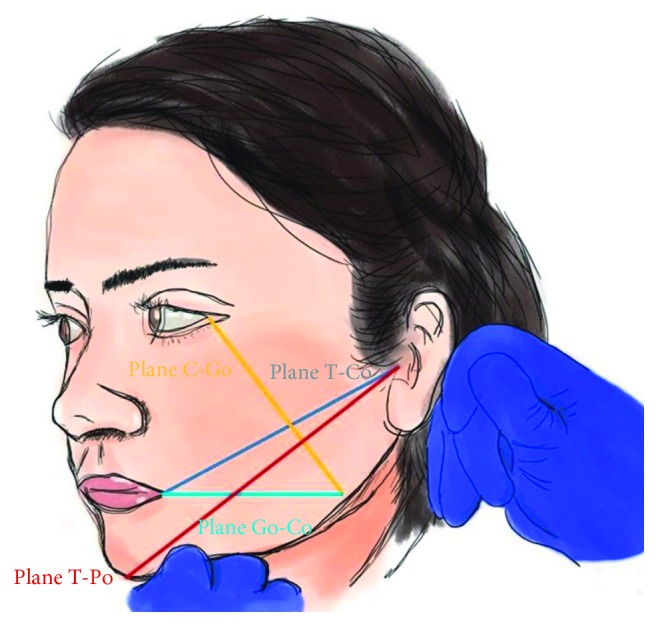
Facial planes for swelling measures.

The facial points pogonion (Pg), labial commissure (Co), gonion (Go), outer eye corner (C), and tragus (T) were marked with indelible ink; measurements of swelling were performed with a millimetric ruler from the facial planes (T-Co, T-Po, C-Go, and Go-Co). The inflammation variable comprised the sum of all of the planes described previously (Figure 2). An independent examiner performed the measurements at the following different times: baseline/time 0 (prior to the surgery); time 24 (24 h after the surgery); time 48 (48 h after the surgery), and time 72 (72 h after the surgery).

Trismus was measured with a millimetric ruler, asking the patient for the maximal oral opening possible and recording the distance from the incisal edge of the upper central incisors to the incisal edge of the lower central incisors. Clinical and radiographic variables recorded prior to the surgery are described in Table 1.

All patients were evaluated at 24, 48, and 72 postoperative hours. For swelling response as a dynamic process, a Mixed Repeated Measures Model (MRMM) analysis was employed. This model included all measurement times (random component), together with different variables (fixed component) (Table 1), and determined which variables result with significance in the swelling and trismus processes during the 72 h follow-up. Statistical software R ver. 3.2.3 was used, with the following packages: BBconRR, car, ggplot2, nlme, reshape, and Rcmdr, with 95% confidence intervals (95% CI).

## 3. Results

Forty-five patients were analyzed during a 72 h follow-up period. The baseline characteristics of the sample are described in Table 2.

For the swelling and trismus variables, the multivariate MRMM, restricted maximal likelihood (REML), and backward-stepwise methods were used. The initial model included all of the variables in the fixed component described in Table 1, and, in the random component, all times were included and each patient was measured 3 times, for a total of 135 measurements. For the swelling model, we used a “varIdent” variance structure [16]; this model was compared with the null model (only intercept included) and this comparison showed *p* value < 0.05. Therefore, and based on the likelihood test, we determined the maximal parsimony model and deleted no significant terms in each iteration; the final model included the significative variables (*p* < 0.05): GENDER, AGE, BMI, and RRB, and the estimates and the significant values are shown in Table 3.

For the trismus variable in the MRMM, the same previous methods were used, and the variance structure utilized was “varPower” [16]; the fixed component included all variables presented in Table 1, and the random component included all trismus measures. The model was compared with the null model (only intercept included), and this comparison showed *p* value > 0.05. Thus, the variables included in the fixed component do not explain the changes in the trismus measures.

## 4. Discussion

To understand clinical and radiographic characteristics as predictor factors of swelling and trismus, after mandibular third molar surgery, it is important to recognize the risk factors associated with clinical complications. Several studies have measured the difficulty of this surgical procedure and clinical and radiological factors [1, 11–14, 17, 18].

Swelling has been determined by different techniques described in the literature; these techniques include visual inspection [6], facial points [19], photographic techniques [20], Computed Tomography (CT) [21], and others [7, 15, 22]. In the present study, swelling was estimated by the sum of the four facial planes. The advantages of this method are its simple implementation and low cost. Trismus was determined by maximal opening of the mouth and the distance between the lower and upper incisors.

Gender, body mass index (BMI), relation to lingual and buccal walls, and age of the patients were determinants in explaining the swelling measurements. The importance of gender in facial swelling was found in the present study, swelling was higher in males than in females, this agreement with that reported by Yuasa and Sigiura [15]. These authors, with a total of 140 patients (153 surgical procedures, 64.7% females), used a sum of two planes (T-Co and C-Go) divided by 2, to measure facial swelling. Measurements were made on postoperative days 1 and 7. The statistical analyses used in their study included logistic regression and correlation without multiple measurements, and the difference between both sexes in the swelling was observed in the first time. This cannot be extended to the dynamic process of swelling. To deal with this issue, we propose the analysis of variables with the MRMM, which is applicable with the longitudinal design in our study [23].

On the other hand, de Santana-Santos et al. [24] reported the influence of the “gender” of the patients in the prediction of swelling. These authors found higher swelling in females than in males, with a sample size of 80 patients (32.5%, females). They employed five measurements of distance between two facial points (the sum of five measurements divided by 5); the measurements were made on postoperative days 2 and 7, and the statistical analysis used was correlation and group differences in one measurement time. Osunde and Saheeb [25] did not find a significant effect of sex on the swelling variable; these authors recruited a total of 150 patients (56%, female). They used the arithmetic mean of two planes (T-Co and C-Go) and the difference between each postoperative measurement at days 1, 2, 3, 5, and 7 and baseline as swelling measurement of the day. However, the authors found a higher mean facial swelling in females than in males, but this result was not significative. The analysis used in their study was one-way ANOVA.

BMI was also reported by de Santana-Santos et al. [24]; they did not find an association with the prediction of swelling. In their study, the authors categorized the BMI value and the swelling measurement. BMI is a measurement of body fat based on height and weight; adipose tissue is a major contributing factor to systemic inflammation, generating approximately one third of the circulating proinflammatory cytokine InterLeukin (IL)-6 [26]. The association of BMI and swelling has been previously explained; people with obesity often experience higher concentrations of inflammatory biomarkers than their normal-weight counterparts [26, 27]. In the present study, the BMI variable had a low weight to explain the swelling in the patients (Table 3).

The relation of lower third molar with lingual and buccal walls has not been, to our knowledge, reported previously as a predictor factor of swelling. Several studies have reported the radiographic classification as a factor contributing to the complexity of the surgery [6, 13, 17, 28, 29]. In the present study, the proximal relation of the lower third molar to the lingual wall, or the relation in the middle position between buccal and lingual walls, corresponded to an increase in the swelling measures in the postoperative evaluation; this can be explained by the hard tissue removed. When the third molar is buccally erupted, the total bone removed for the surgical extraction is lower; on the other hand, when the third molar is in lingual position, the amount of bone removed increases.

The age of the patients has been reported as a predictive factor for swelling. Olmedo Gaya et al. [30] reported increased swelling with increased age. According to a report by Bello et al. [29], there is a positive association between age and swelling and age and trismus. Finally, Osunde et al. [12] did not show a significant association between age and swelling in mandibular third molar surgery. In the present study, the age variable shows an inverse relationship with swelling; this implies that if the patient's age increases, the swelling decreases; however, this relationship shows low weight to explain the swelling (Table 3). This relationship can be explained by the changes involved in the immune system associated with the inflammatory response related to age [31].

One of the advantages of this study is the multiple measurement period. Because the swelling process is dynamic and the main changes are present in the first 72 h [9], the MRMM can be adjusted for multiple measurements and used in a longitudinal approach, and the methods control the intra- and interpatient variability when repeated measures design is used and have a more flexible structure for the analysis [23, 32]. The repeated measures design increased the number of measurements on the same patient; this means we can use more repetitions or measurement values in the model with the same number of patients. However, this type of analysis includes some limitations: the slope values are only valid in the range of the variables included; out of this range, this model cannot be applied. Moreover, the swelling measure was linear and not volumetric and did not involve the volume of the change of tissue. van der Meer et al. [22] and Yamamoto et al. [21] propose new methods to measure facial swelling, and the use of stereophotogrammetry, CT, and laser surface scanning comprises the principal elements in these procedures. The advantages of this include the reproducibility of the measures, in terms of volume and precision; however, cost and implementation must be taken into account in the planning of the clinical trials.

Further studies are necessary to confirm these findings, with a higher sample size and variable ranges. We suggest the use of MRMM to analyze the data with a longitudinal approach and control the follow-up measurements during the first 72 hours. The facial planes method to measure the swelling can be useful; however, this method has some limitations, and other alternatives can be validated to this endpoint.

## 5. Conclusions

This study suggests the association of male gender, the relation to the lingual and buccal walls, BMI, and age with the swelling measurement. The trismus variable did not show any relationship with explanatory variables.

## Figures and Tables

**Table 1:** Variables included in the model (fixed component).

Code	Variable	Units
Gender	Gender of the patient	F (female)
		M (male)
Baseline	Basal value (time 0)	mm
Age	Age of the patient	Years
BMI	Body mass index	kg/m^2^
TQX	Surgical elapsed time	Minutes
Total intake	Total consumption of analgesic intake in 72 hours	Number of tablets
R2M	Relationship of the third molar to second molar	0: Crown directed at or above the equator second molar
		1: Crown directed below the equator second molar
		2: Crown/roots directed to the middle of the second molar
		3: Crown/roots directed to the apical third of the second molar
RRM	Relation to the mandibular ramus	0: Sufficient space in the dental arch
		1: Partially impacted in the ramus
		2: Completely impacted in the ramus
		3: Completely impacted in the ramus in distoangular position
RRA	Relation to the adjacent alveolar crest (from the uppermost point of the tooth)	0: Completely erupted
		1: Partially impacted, but widest part of the crown (equator) is above the bone
		2: Partially impacted, but widest part of the crown (equator) is below the bone
		3: Completely impacted
RRB	Relation to the lingual and buccal walls	0: Closer to buccal wall
		1: In the middle between lingual and buccal walls
		2: Closer to lingual wall
		3: Closer to lingual wall, when the tooth is partially/completely impacted

**Table 2:** Basal characteristics.

Variable	*n*=45	Min-max
Age (mean (sd))	23.58 (3.34)	18–29
Gender = male (%)	11 (24.4)	—
BMI (mean (sd))	23.90 (3.24)	18.87–32
TQX (mean (sd))	19.02 (5.38)	7–28
Total intake (mean (sd))	4.8 (2.61)	0–9
R2M (%)		
0	3 (6.7)	
1	19 (42.2)	
2	23 (51.1)	
RRM (%)		
0	1 (2.2)	
1	31 (68.9)	
2	13 (28.9)	
RRA (%)		
1	16 (35.6)	
2	27 (60.0)	
3	2 (4.4)	
RRB (%)		
0	2 (4.4)	
1	31 (68.9)	
2	12 (26.7)	

**Table 3:** Estimates of the significant variables for swelling response.

Variable	Slope value	CI 95%	*P* value	Eta^2
Gender (male)	40.99	28.6,53.4	<0.000001	0.2613
BMI	3.06	1.4, 4.8	0.00027	0.0841
RRB	*RB1* 38.82	14.8,62.8	0.00143	0.0646
	*RB2* 44.74	19.7, 69.7		
Age	−2.57	−2.6, −1.0	0.00068	0.0591
Residual	—	—	—	0.5309
